# Alkaline Dentifrice

**Published:** 1859-04

**Authors:** 


*Alkaline Dentifrice.*—Powder of dried bone, 80 parts,

| | | |
|---|---|---|
| Precipitated chalk, | 20 | " |
| Bicarbonate of soda, | 5 | " |
| Powdered orris root, | 2 | " |

It should be used every night after brushing the teeth. It is of especial use to delicate persons whose teeth easily spoil, to pregnant women, and in the course of diseases which render the buccal fluids acid. After using it the teeth must not be too much washed, so that a little may remain in the intestines, and thus neutralize the acid as it appears. So says the *Bulletin de Therapeutique*, and we are inclined to coincide in its recommendation.

